# Commentary: Tauroursodeoxycholic acid regulates macrophage/monocyte distribution and improves spinal microenvironment to promote nerve regeneration through inhibiting NF-κB signaling pathway in spinal cord injury

**DOI:** 10.3389/fphar.2025.1620107

**Published:** 2025-06-09

**Authors:** Yi Chen, Lei Wu, Ruijie Ma

**Affiliations:** ^1^ Department of Neurobiology and Acupuncture Research, Key Laboratory of Acupuncture and Neurology of Zhejiang Province, The Third School of Clinical Medicine (School of Rehabilitation Medicine), Zhejiang Chinese Medical University, Hangzhou, China; ^2^ Department of Acupuncture, The Third Affiliated Hospital of Zhejiang Chinese Medical University, Hangzhou, China

**Keywords:** tauroursodeoxycholic acid, macrophage, spinal cord injury, spinal microenvironment, NF-κB

## Introduction

We carefully read the study published by Hou et al. in Front. Pharmacol, which explored how tauroursodeoxycholic acid (TUDCA) regulates macrophage/monocyte distribution and improves spinal microenvironment to promote nerve regeneration through inhibiting NF-κB signaling pathway in spinal cord injury (Hou et al., 2025). This study aims to investigate the effect of TUDCA on macrophage/monocyte infiltration and distribution, scar formation, NSCs proliferation and migration, and nerve degeneration during the first 7 days to explore the underlying mechanism of TUDCA in SCI. Through *in vitro* experiments (such as Transwell analysis, BrdU staining, TUNEL staining) and *in vivo* experiments (such as SCI mouse model, RNA sequencing, immunofluorescence staining), the authors not only confirmed the neuroprotective effect of TUDCA, but also combined immune cell regulation with microenvironment improvement. We believe that this study is of great significance for spinal cord injury research.

We would like to offer a few suggestions that could further strengthen the study’s reliability and completeness.

## Enhancing TUDCA mechanism validation and safety profiling in SCI studies

Preclinical studies have shown that TUDCA works not only by regulating and inhibiting the apoptotic cascade, but also by reducing oxidative stress, protecting mitochondria, producing anti-inflammatory effects, and serving as a chemical partner to maintain protein stability and correct folding (Khalaf et al., 2022). Therefore, the author established a TUDCA group to verify the intervention effect of tauroursodeoxycholic acid on the pathological process of SCI and observe its effects on nerve regeneration and inflammatory response. We suggest evaluating the safety and baseline effects of drugs under non-invasive conditions, setting up normal mice or cell groups that only receive TUDCA treatment, and conducting WB and fluorescence experiments, which can help rule out potential effects of drugs on non pathological states.

## Multi dimensional regulation of spinal cord microenvironment by TUDCA

In addition, although the author used RAW264.7 mouse monocyte macrophage line and primary spinal cord neural stem cell NSCs, other cell types related to the spinal cord microenvironment were not included. Microglia and astrocytes are pivotal in neuroinflammation and neural repair. Including these cells will clarify TUDCA’s role in modulating glial responses and its indirect effects on NSCs/macrophages. We suggest that new experiments using primary microglia and astrocytes isolated from rodent spinal cord tissue to evaluate TUDCA’s effects on these cells. These experiments will include: Functional assays (e.g., phagocytosis, cytokine secretion in microglia; reactive astrogliosis markers in astrocytes). Cross-talk analysis via co-culture systems (e.g., microglia-NSC or astrocyte-macrophage interactions) to mimic *in vivo* conditions.

## Functional validation of NF-κB signaling via gene knockout to elucidate TUDCA’s role in spinal cord injury nerve regeneration

Finally, although the author found through GO analysis and GSEA that TUDCA regulates inflammation and development related pathways, functional validation of the key differentially expressed gene NF-κB pathway was not conducted. We suggest that the author knock out the NF-κB differentially expressed gene to further validate the experimental results. This will provide a more comprehensive understanding of the mechanism of NF-κB signaling pathway in regulating nerve regeneration in spinal cord injury.

## Discussion

We commend the authors’innovative work and believe that their findings greatly deepen our understanding of TUDCA in spinal cord injury repair. This study provided evidence that TUDCA treatment regulated monocyte/macrophage distribution and improved the microenvironment to promote nerve regeneration in SCI mice. We look forward to further exploring TUDCA and its regulatory mechanisms in this context.
